# Muscle Tissue Quality of Raw and Sous-Vide Cooked Wild and Farmed Pikeperch

**DOI:** 10.3390/foods11233811

**Published:** 2022-11-26

**Authors:** Monika Modzelewska-Kapituła, Renata Pietrzak-Fiećko, Krzysztof Kozłowski, Mirosław Szczepkowski, Zdzisław Zakęś

**Affiliations:** 1 Department of Meat Technology and Chemistry, Faculty of Food Sciences, University of Warmia and Mazury in Olsztyn, Plac Cieszyński 1, 10-719 Olsztyn, Poland; 2 Department of Commodities and Food Analysis, Faculty of Food Sciences, University of Warmia and Mazury in Olsztyn, Plac Cieszyński 1, 10-719 Olsztyn, Poland; 3 Department of Ichthyology and Aquaculture, Faculty of Animal Bioengineering, University of Warmia and Mazury in Olsztyn, 10-719 Olsztyn, Poland; 4 Department of Sturgeon Fish Breeding, Stanisław Sakowicz Inland Fisheries Institute in Olsztyn, 10-719 Olsztyn, Poland; 5 Department of Aquaculture, Stanisław Sakowicz Inland Fisheries Institute in Olsztyn, 10-719 Olsztyn, Poland

**Keywords:** freshwater fish, thermal treatment, nutritional value, fatty acids, colour

## Abstract

The aim of the study was to compare the chemical and fatty acid composition, colour, and sensory quality of wild and farmed pikeperch. Raw wild pikeperch had a higher moisture and ash contents, as well as pH value, but lower fat and protein contents than farmed pikeperch. In sous-vide fillets, a higher fat and a lower protein content were noted in farmed fish. Slight differences in colour attributes between farmed and wild fish affected neither chroma nor hue in raw and sous-vide fillets. Wild and farmed fish fillets prepared using sous-vide were scored similarly in the sensory assessment. Although wild fish had a more beneficial fatty acid composition demonstrated by a higher proportion of polyunsaturated fatty acids, including eicosapentaenoic and docosahexaenoic, a higher fat concentration in farmed pikeperch resulted in obtaining similar or even higher concentration of these fatty acids in sous-vide fillets. Regardless of the origin of pikeperch it might be recommended as a valuable part of a daily diet.

## 1. Introduction

Catches of pikeperch (*Sander lucioperca*) have remained fairly stable at approximately 20,000 tons annually over the past 40 years [1]. Increasing demand for this species (primarily in Western European countries), the muscle tissue of which has a high nutritional quality, could potentially be met by aquaculture. Until recently, pikeperch was cultured exclusively in earthen ponds mainly in the temperate zone. This type of pikeperch production technology is extensive, and the rearing cycle is long, running from three to five years [2]. Consequently, while the scale of pikeperch pond production is small, it does exhibit an increasing trend [1]. Substantial increases in pikeperch production have been noted in the past 10 to 15 years (and especially after 2010) with the development and implementation of technologies for producing this species in recirculating aquaculture systems (RAS). This is a new and dynamically developing technology, and pikeperch is viewed as a very promising species in the context of further technological developments [3]. When optimal temperatures for pikeperch growth are applied, culturing this species in RAS reduces the production cycle to 13–15 months [4]. Pikeperch reared in RAS are fed high-energy formulated feed that determines the meat tissue proximate composition (usually increased lipid contents), dietary value (e.g., the fatty acids profile), and organoleptic attributes [5]. Research indicates that supplying this type of diet to percids can advantageously influence dietary values of the meat by increasing concentrations of polyunsaturated fatty acids (PUFA), which are particularly valuable in human nutrition [6].

In European culinary tradition, fish are consumed after thermal treatment (cooking, frying, hot smoking) or salting and acid marinating. Each of these processes results in products with distinct sensory properties and nutritional value. In households and restaurants, most commonly used thermal processes are cooking and frying. However, a high-temperature treatment might negatively affect the nutritional profile of fish products. Bouriga et al. [7], who applied hot smoking to increase a consumer acceptability of pikeperch fillets, noted that it decreased PUFA proportion, which is vitally important from a nutritional perspective, and promoted lipoperoxidation and lipid oxidation in the fillets. Easy to conduct in household conditions, pan-frying generally produces higher changes in the fish lipid composition than other cooking methods, which are manifested in higher losses of DHA (docosahexaenoic) and EPA (eicosapentaenoic) compared to other cooking methods [8]. Sous-vide is a relatively new culinary technique, which is considered the best method for the preservation of bioactive compounds present in a raw material during cooking. In this technique, heating is proceeded at a relatively low temperature (in the case of fish, it is even below 70 °C), under vacuum conditions for longer time than other traditional methods [9]. Moreover, sous-vide treatment is effective as a method for extending the shelf-life of cooked products by reduction of aerobic bacteria growth and preventing foods from recontamination [9]. Bearing this in mind, in the present study, we used sous-vide as a method for the thermal treatment of wild and farmed pikeperch fillets. Due to the scarcity of information about differences in the quality of farmed and wild pikeperch as a raw material and final products for human consumption, the aim of the study was to compare wild and farmed pikeperch in terms of the chemical composition, including fatty acid proportion and concentration, colour, pH, expressible water, and the sensory quality.

## 2. Materials and Methods

### 2.1. Fish, Origin, Rearing Conditions

Effect of pikeperch origin (farmed vs. wild) was analysed using 12 farmed (age 2+, body weight 1173.4 ± 120.4 g, total length 47.8 ± 1.9 cm) and 12 wild fish (age 4+ (determined from scale readings; Brylińska [10], body weight 1234.3 ± 178.0 g, total length 51.3 ± 2.1 cm). The farmed fish, which were intensively reared with formulated feed (in recirculating aquaculture system, RAS) originated from the Department of Sturgeon Fish Breeding of the Inland Fisheries Institute in Olsztyn, Poland. Larval and juvenile pikeperch were reared in RAS according to methods developed previously [2,11]. The larvae were reared in tanks with a volume of approximately 1.0 m^3^. After the fish obtained a body weight of 25 g, they were moved to larger rearing tanks of approximately 2 m^3^. In the last 12 months (fattening phase), fish were fed Aller Rep Ex (Aller Aqua, Christiansfeld, Denmark). The composition of the feeds was: crude protein—53.0%, crude fats—14.0%, nitrogen free extract (NFE)—14.5%, crude fibre—1.3%, and gross energy—20.9 MJ kg^−1^ feed. Fatty acid composition of the feed is presented in Table 1. The fish were fed using an automatic band feeder (Fischtechnik GmbH, Nienburg, Germany) that delivered feed continuously for 16 h d^−1^. Water temperature during fattening phase ranged from 16.8 °C to 22.7 °C. The oxygen concentrations at the rearing tank’s outflow did not fall below 6.1 mg O_2_ L^−1^. The concentration of total ammonia nitrogen (TAN = NH_4_^+^-N + NH_3_-N) measured at the rearing tank outflows did not exceed 0.4 mg TAN L^−1^ and that of nitrites (NO_2_-N) did not exceed 0.2 mg NO_2_-N L^−1^. The water pH was within the range of 7.78–8.08. The stocking density in the last months of rearing was approximately 30 kg m^−3^.

The wild specimens were caught in November in Lake Wymój (Mazurian Lake District, Northern Poland). Gill nets were used (mesh size length—55, 70, 80 mm). The water temperature was 6.0 °C when the fish were caught. The prey fish of wild pikeperch (roach, *Rutilus rutilus*, and perch, *Perca fluviatilis*) were captured at the same time (in the same lake using gill nets with mesh size 18, 20, 22, 25 mm). They were ground together, and their composition was determined as: 4.8% ash, 0.6% fat, and 76.5% moisture. Immediately following capture, the pikeperch were killed and kept on ice until processing (not longer than 6 h). Fillets for the study were obtained after gutting the wild and farmed pikeperch, de-heading with a simple cut, fin removal, filleting, and skinning the fillets (Table 1).

### 2.2. Experimental Design

The fillets were transported on ice to a laboratory of Faculty of Food Sciences; one fillet from each fish was used to determine the quality of raw tissue, whereas the other one was subjected to thermal treatment and then to chemical and sensory analyses. In raw fillets, colour, pH, moisture, fat, protein, ash contents, fatty acid composition, and expressible water content were determined, whereas in cooked fillets, the following attributes were determined: colour, cooking loss, moisture, fat, protein, ash contents, fatty acids composition, and sensory analysis. Before thermal treatment, each filet was divided into two separate parts (after the thermal treatment, one was used in the sensory analysis, while the other was used to determine the chemical composition). Samples for thermal treatment were weighted and individually vacuum packed in plastic punches suitable for sous-vide cooking, and then they were heated in a sous-vide device at a temperature of 65 °C for 40 min. After that, the samples for sensory analysis were evaluated, whereas the remaining were chilled in cold water with ice and refrigerated at a temperature of 4 °C overnight and then used in chemical analyses.

### 2.3. Colour Measurements

Colour parameters (CIE L*a*b*) were measured on the surface of the muscle tissue of raw and cooked fillets using Konica Minolta CR-400 (Sensing Inc., Tokyo, Japan) (D65 illuminant, 2° standard observer angle). CIE L* a* and b* values were measured in triplicate in the dorsal part of the fillets on their inner side. L* values indicate the lightness (from 0 black to 100 white), a* the proportion of red (a* > 0) or green (a* < 0) hues and b* the proportion of yellow (b* > 0) or blue (b* < 0) hues. Using the following equations, values of chroma (C) and hue angle (h) were calculated: C = (a*^2^ + b*^2^)^0.5^ and h = atan (b*/a*)·360°/(2·Π) [12].

### 2.4. pH and Expressible Moisture Determination

Values of pH and expressible water content were determined in the raw muscle tissue of fish. Values of pH were measured directly in three different locations of each raw fillet with a HI 8314 pH-meter equipped with an FC 200 combined electrode (Hanna Instruments Polska, Olsztyn, Poland). Prior to measurements, the pH-meter was calibrated (pH 7 and pH 4 buffers). Expressible moisture was determined in triplicate for each sample according to the method described by Benjakul et al. [13] with a slight modification [14].

### 2.5. Moisture, Fat, Protein, and Ash Contents

Raw and sous-vide fillets were individually ground twice through a 4 mm mesh. To determine the moisture content, samples were dried at a temperature of 103 ± 2 °C [15]. After dying, they were used to determine fat content according to the Soxhlet method [16] with ethyl ether as a solvent. The content of crude protein was determined according to the Kjeldahl method with a 6.25 multiplier, and the ash content by sample mineralization at 550–600 °C [17]. The analyses were performed in duplicate.

### 2.6. Fatty Acids

Fatty acid proportions and contents were determined using the method described in detail in Modzelewska-Kapituła et al. [14]. Briefly, muscle fat was extracted according to Folch et al.’s [18] method using a mixture of chloroform and methanol (2:1 *v*/*v*). Methylation of fatty acids was conducted using a chloroform–methanol–sulphuric acid (100:100:1) mixture [19]. Chromatographic separation was done using 7890A Agilent Technologies gas chromatograph equipped with a flame-ionization detector (FID) and a 30 m 0.32 mm internal diameter fused silica capillary column (matrix active group: poly(ethylene glycol)phase, Supelco, Bellefonte, PA, USA). Supelco (Bellefonte, PA, USA) standards were used to identify a particular fatty acid by comparing retention times. The results were presented as relative percentage (% total fatty acids) and content (mg/100 g wet tissue) of fatty acids in raw and sous-vide cooked fillets. The content of a particular fatty acid in fish muscle tissue was calculated separately for each sample based on fat content and coefficient for lean fish (0.70) [20].

### 2.7. Sensory Analysis

The analysis was carried out according to Modzelewska-Kapituła et al. [21]. Immediately after sous-vide cooking, samples for sensory evaluation were prepared (dimensions approx. 2.0 cm × 2.0 cm). Samples were assessed by a 6-member sensory panel, the members of which were chosen, trained, tested, and monitored according to ISO guidelines [22]. Each panellist assessed all samples obtained from every fish, cut from the same fillet part. Four separate sessions of sensory evaluation were carried out; during each session, 6 samples were evaluated. Samples were evaluated using a 1 to 5 point scale, in terms of colour (from 5—homogeneous, typical, to 1—very inhomogeneous), aroma (from 5—typical, clearly perceptible, to 1—strong foreign), texture (from 5—firm, particularly tender, to 1—very soft or very hard, fibrous), juiciness (from 5—very juicy, to 1—very dry), and taste (from 5—desirable, typical, intense, no foreign aftertastes, to 1—atypical, strong foreign aftertaste) [21].

### 2.8. Statistical Analysis

Normal distribution of data and variance homogeneity were tested using Shapiro–Wilk’s and Leven’s tests, respectively. Data showed a normal distribution and variance homogeneity; therefore, to compare mean values, one-way variance analysis was applied (Statistica 13.3, Tibco Software Inc., Palo Alto, CA, USA).

## 3. Results and Discussion

### 3.1. Comparison of Weights and Yields of Wild and Farmed Pikeperch

Parameters of wild and farmed pikeperch are shown in Table 1. Wild fish had a significantly longer body than farmed ones, but there were no differences in body weight, gutted fish, and skinned fillet weights. The results obtained in the present study resemble the findings of Jankowska et al. [23] who studied differences between wild and farmed pikeperch.

A higher proportion of skinned fillets in relation to body weight was found in wild pikeperch, which might indicate their higher economic efficiency and might result from a longer body. Nevertheless, it should be highlighted that farmed pikeperch at the age of approx. 2 years reached a similar body weight to wild 4-year-old pikeperch. This resulted from differences in feed composition (commercial feed vs. prey fish) and water temperatures (lake vs. RAS). The feed also differed in fatty acid composition (Table 2).

Significant differences were noted in each fatty acid proportion, as well as the sum of particular fatty acid groups. The wild pikeperch fodder had higher saturated and polyunsaturated fatty acid (SFA and PUFA, respectively) and n-3 fatty acids proportions but lower monounsaturated fatty acids (MUFA) and n-6 fatty acids than farmed fish fodder.

### 3.2. The Quality of Raw Wild and Farmed Pikeperch Fillets

The chemical composition of raw fillets obtained from wild and farmed pikeperch is shown in Table 3.

The fillets from wild pikeperch had a higher moisture (78.9%) and ash (1.2%) contents and pH (6.7), but a lower fat (0.4%) and protein (19.3%) content than farmed pikeperch (76.7%, 1.1%, 6.5, 1.1%, and 20.8% for moisture, ash, pH, fat, and protein, respectively). These differences in the chemical composition resulted from differences in the feed of wild and farmed fish, including the fat content which was 0.6% and 14% in wild and farmed pikeperch fodder, respectively. Similar to results of the present study, a higher fat content in farmed vs. wild pikeperch was noted by Jankowska et al. [23]. This might be attributed to a higher fat content in farmed pikeperch feed, but also to different life conditions—food availability, mobility, and energy demand. Generally, the wild and farmed fish used in the present study had a lower fat content than that noted by other authors. Bouriga et al. [7] reported that the fat content in wild pikeperch was 1.9%, whereas protein was 17.7% and ash was 1.1%. A higher protein content, similar to that noted in the present study, was found by Çağlak and Karsli [24] in wild pikeperch (from 17.8% to 19.4% regarding of the season, for spring and autumn, respectively). These differences in the chemical composition between the results of the present study and those obtained by other authors in terms of wild fish might be caused by multiple factors such as the living environment, food availability, and a fishing season [24]. The results of the present are slightly different from those obtained by Ćirković et al. [25] for farmed pikeperch. They reported the following chemical composition: protein 19.3%, fat 0.4%, moisture 79.3%, and ash 1.0%, which are lower in terms of fat and protein contents than the results of our study and might be produced by differences in fodder offered to fish (natural food produced with benthic and planktonic organisms in Ćirković et al. [25] and high-energy formulated feed in the present study). The harvest time did not affect the results because fish in both studies were caught in autumn (October and November in Ćirković et al. [25] and the present study, respectively). The results of the proximate composition of farmed pikeperch were similar to those reported by Ljubojević et al. [26] for farmed pikeperch available in the Serbian market; however, the fat content (1.8%) was lower than in the present study.

In terms of colour, wild and farmed fish differ only in redness (a*), which was higher in wild pikeperch (Table 3). Higher a* values in the fillets of wild fish compared to farmed were also noted by González et al. [27] in yellow perch (*Perca flavescens*) and might be attributed to multiple factors such as lower fat content, blood vasculature, higher deposition of melanin due to dietary effects, or enzymatic reactions from tyrosine. Despite differences in pH values, there were no differences in expressible water contents (Table 3). Similar findings were noted in a previous study conducted using wild and farmed northern pike (*Esox lucius*) [14]. The expressible water is a measure of the ability of muscle tissue to hold moisture and is regarded as an important technological feature. Lower ability to hold moisture is manifested in an excessive drip loss in the package, which in turn decreases the attractiveness of packed fresh fish and their fillets and negatively affects fish tissue texture [28]. Both the ability of muscle tissue to hold moisture and colour are affected by pH value [28,29]. The results obtained in the present study thus indicate that although there were significant differences in the pH value of farmed and wild fillets, they were too small to affect the expressible water content and the same water holding capacity, as well as the chroma and hue of wild and farmed pikeperch fillets. Table 4 contains values obtained for each fatty acid detected in fat extracted from the muscle tissue.

Significant differences in fatty acid composition and concentration in the muscle tissue of wild and farmed pikeperch were noted. Generally, the proportions of SFA, MUFA, and PUFA in the wild pikeperch were similar and accounted for 32 to 35% of total fatty acids. The most prevailing fatty acids were C16:0 (palmitic acid) and C18:1cis9 (oleic acid), which accounted for 22% and 17% of total fatty acids, respectively. In farmed fish, a higher proportion of MUFA was noted in comparison to SFA and PUFA (52% of total fatty acids vs. 20% and 28%, respectively). The most abundant fatty acids were similar to those noted in wild pikeperch, but in different proportions—C16:0 accounted for only 15% and C18:1cis9 for as much as 36% of total fatty acids (Table 4). The abundance of palmitic acid (C16:0) and oleic acid (C18:1n-9) in pikeperch fat was also found by Bouriga et al. [7]. Fatty acid proportions noted in the present study slightly differ from the results obtained by Çağlak and Karsli [24], who noted that PUFA’s proportion accounted for 43.3% of total fatty acids, whereas MUFA’s accounted for 19.6% in wild pikeperch, and the study by Jankowska et al. [23], who reported the following fatty acid composition in wild pikeperch: 27.8% SFA, 21.4% MUFA, and 50.8% PUFA. Differences between wild and farmed pikeperch in fatty acid composition were noted in terms of MUFA and PUFA by Jankowska et al. [23]. In their study, wild pikeperch had a significantly lower MUFA and higher PUFA than farmed fish receiving an artificial feed. However, no differences were found in n-3 and n-6 fatty acids [23]. The differences between the results obtained in the present study and reported by Jankowska et al. [23] result from different fatty acid compositions of the feed used in those studies and indicate the role of the feed in shaping the fatty acid composition of fish muscle tissue. This is supported by the results of Ćirković et al. [25] who reported a fatty acid composition (SFA%, MUFA%, and PUFA%) in farmed pikeperch fed natural fodder similar to those obtained in the present study in wild pikeperch. Interestingly, in the present study, in farmed fish fat additional fatty acids such as C22:1 n-11 and C22:1 n-9 were detected, which were not present in the fat extracted from wild pikeperch fillets. This was a result of the presence of those fatty acids in the feed of farmed pikeperch.

Jankowska et al. [23] showed that the content of DHA in the farmed pikeperch lipids was twice as high as that in the artificial feed and four times that in the natural food offered. In the present study, the proportion of DHA increased in muscle tissue of wild and farmed pikeperch, but the increase was not so high (about 30% in wild and 9% in farmed pikeperch in respect to the feed). The increase in DHA in muscle tissue might be explained by the desaturation of native forms of polyunsaturated acids taken up by the fish from the feed. The process involves the introduction of double bonds into a molecule by desaturases Δ6, Δ5, Δ4, and chain elongation, mediated by elongases. Jankowska et al. [23] concluded that shorter chain n-3 acids (C18) in the feed are elongated and desaturated in the pikeperch body, and as a result, longer-chain polyunsaturated acids, mainly DHA, are formed.

Differences in fatty acid proportions and a higher content of fat in farmed pikeperch produced differences in the concentration of particular fatty acids in fresh fillets (Table 4). Although the proportion of PUFA (valuable from a nutritional perspective), including n-3 fatty acids, was higher in wild fish, the farmed fish was a better source of those acids due to their higher concentration (mg/100 g). Nevertheless, farmed fish contained a higher amount of SFA and MUFA than wild ones. The n-6/n-3 ratio was lower in wild pikeperch, although in both studied fish it was low and beneficial from a nutritional perspective. Çağlak and Karsli [24], who studied the quality of pikeperch caught in Beyşehir Lake (Turkiye) in four different seasons, found that the amount of PUFA (89.85–109.11 mg/100 g) was higher than SFA (55.08–81.89 mg/100 g) and MUFA (29.16–78.89 mg/100 g). The results for farmed pikeperch obtained in the present study are similar to those reported by Çağlak and Karsli [24]; however, in farmed fish, a higher concentration of MUFA than PUFA and SFA was found.

### 3.3. The Quality of Cooked Wild and Farmed Pikeperch Fillets

There were slight differences in the quality of cooked fillets between wild and farmed pikeperch. In terms of the chemical composition, the fish differed in fat content, which was higher in farmed pikeperch, and protein content, which was higher in wild fish fillets (Table 5). The moisture content in sous-vide pikeperch noted in this study was similar to that reported by Gladyshev et al. [30] for pikeperch cooked using different cooking methods (boiling and stewing in water and in a convection steam oven), which ranged from 75.6% to 79.4%. Although farmed fillets showed a lower average cooking loss than wild pikeperch fillets (20% vs. 22%), the difference was not significant (Table 5).

Fillets differed also in colour—those obtained from farmed pikeperch were lighter and had a higher redness (a* values). A higher lightness of sous-vide farmed pikeperch fillets corresponded with a significantly higher fat content in those samples, which is a known factor affecting muscle tissue [31]. However, the sous-vide fillets did not differ in chroma or hue and thus would be similarly perceived by consumers. In terms of the sensory quality, the fillets did not differ and were scored similarly (Table 5). This finding indicates that from a sensory point of view, both fish types are similarly suitable for preparing dishes.

As was noted in the raw fillets, the sous-vide cooked fillets obtained from wild and farmed pikeperch differed in fatty acid composition (% of total fatty acids) and concentration (mg/100 g). Although wild pikeperch fillets had a higher proportion of the sum of PUFA and n-3 fatty acids, sous-vide cooked fillets obtained from farmed fish were a better source of those fatty acids in the human diet due to higher concentrations of these components (Table 6). Generally, a fatty acid concentration (a sum of SFA, MUFA, and PUFA) noted in sous-vide wild pikeperch (3.6 mg/g of wet tissue) was similar to that reported by Gladyshev et al. [30] for boiled (3.7 mg/g) and stewed (3.4 mg/g) wild pikeperch. The n-6/n-3 ratio noted in their study in boiled and stewed pikeperch were also similar to that noted in the present study (0.4), which is a low value and beneficial from a nutritional point of view [32]. In farmed sous-vide pikeperch fillets, the ratio was higher but still low (1.0, Table 6); therefore, sous-vide pikeperch obtained from both a lake and a farm is a valuable food product. This is supported by similar EPA and DHA concentrations in sous-vide fillets of wild and farmed pikeperch (Table 6). The consumption of fish rich in these fatty acids might result in a decrease in the n-6/n-3 ratio of a diet and prevent many chronic diseases connected with inflammatory processes, such as cardiovascular diseases, inflammatory bowel disease, cancer, and rheumatoid arthritis, as well as psychiatric and neurodegenerative illnesses [32].

## 4. Conclusions

In this study, wild and farmed pikeperch were compared in terms of yields, pH, colour, and chemical composition of raw muscle tissue, as well as chemical composition, colour, and sensory quality of fillets after sous-vide cooking. The weight of wild and farmed pikeperch was similar, but wild pikeperch had a longer body and showed a significantly higher skinned fillet yield. Differences in the fodder chemical composition as well as life environment resulted in differences in raw fillet colour (wild pikeperch fillets had more red hues), proximate chemical composition, and fatty acid profile. Raw wild pikeperch fillets had lower fat and protein contents than farmed counterparts; however, after sous-vide, fillets from wild pikeperch showed lower fat and higher protein contents than those from farmed pikeperch. Wild fish had a more beneficial fatty acid composition manifested by a higher proportion of EPA and DHA. Nevertheless, due to a higher fat concentration in farmed pikeperch, a similar content of those fatty acids in sous-vide fillets was noted in wild and farmed pikeperch. Moreover, sous-vide fillets from farmed pikeperch had a higher amount of PUFA, including n-3; therefore, they were more beneficial from a nutritional perspective. The colour of sous-vide fillets produced from wild pikeperch were darker and less red than fillets produced from farmed fish; however, this did not affect the results of the sensory colour evaluation. There were no differences in the aroma, texture, juiciness, or taste of sous-vide fillets obtained from wild and farmed pikeperch. High results obtained in the sensory assessment indicate a good eating quality of both sous-vide products regardless of the fish origin. The results of the present study clearly indicate the good quality and high nutritional value of farmed pikeperch; therefore, fish producers might be encouraged to produce pikeperch in RAS as an alternative to wild pikeperch, whose population in the environment is limited.

## Figures and Tables

**Table 1:** Characteristics of fish (mean values ± standard error; *n* = 12).

Parameters	Wild Pikeperch	Farmed Pikeperch	*p* Value
Body weight (bw, g)	1234.3 ± 51.4	1173.4 ± 34.8	NS
Total body length (cm)	51.3 ± 0.6	47.8 ± 0.5	*
Gutted fish (g)	1097.8 ± 44.0	1021.0 ± 28.0	NS
Gutted fish (% bw)	89.0 ± 0.9	87.1 ± 1.2	NS
Skinned fillet (g)	573.2 ± 23.4	519.6 ± 15.0	NS
Skinned fillet (% bw)	46.5 ± 0.7	44.3 ± 0.4	*

* *p* ≤ 0.05, NS—non-significant differences (*p* > 0.05).

**Table 2:** Fatty acid composition of wild (*n* = 3) and farmed (*n* = 3) pikeperch feed (mean values ± standard error).

Fatty Acid (% of Total)	Wild Pikeperch Feed	Farmed Pikeperch Feed	*p* Value
Saturated fatty acids (SFA)			
C 12:0	0.144 ± 0.0004	0.251 ± 0.0021	***
C 14:0	4.85 ± 0.008	2.92 ± 0.027	***
C 15:0	0.805 ± 0.0019	0.253 ± 0.0026	***
C 16:0	15.78 ± 0.03	12.97 ± 0.09	***
C 17:0	0.992 ± 0.004	0.162 ± 0.001	***
C 18:0	3.03 ± 0.04	2.04 ± 0.01	***
C 20:0	0.147 ± 0.005	0.374 ± 0.003	***
Monounsaturated fatty acids (MUFA)			
C 14:1	0.312 ± 0.013	0.030 ± 0.001	***
C 16:1	14.93 ± 0.48	2.85 ± 0.03	***
C 17:1	1.22 ± 0.01	0.30 ± 0.01	***
C 18:1 cis9	15.82 ± 0.13	32.09 ± 0.02	***
C 18:1 cis11	4.54 ± 0.03	2.81 ± 0.02	***
C 20:1 n-9	0.52 ± 0.04	4.53 ± 0.03	***
C 22:1 n-11	ND	4.32 ± 0.06	***
C 22:1 n-9	ND	0.51 ± 0.01	***
Polyunsaturated fatty acids (PUFA)			
C 18:2 n-6	5.51 ± 0.02	17.06 ± 0.03	***
C 18:3 n-6	0.440 ± 0.005	0.098 ± 0.001	***
C 18:3 n-3	5.34 ± 0.02	4.70 ± 0.01	***
C 18:4 n-3	1.798 ± 0.005	0.937 ± 0.003	***
C 20:2 n-6	0.349 ± 0.003	0.326 ± 0.002	**
C 20:3 n-6	0.349 ± 0.014	0.049 ± 0.004	***
C 20:4 n-6	4.198 ± 0.040	0.287 ± 0.003	***
C 20:3 n-3	0.378 ± 0.007	0.160 ± 0.002	***
C 20:4 n-3	1.222 ± 0.012	0.350 ± 0.004	***
C 20:5 n-3 (EPA)	7.04 ± 0.06	3.64 ± 0.02	***
C 22:5 n-6	0.846 ± 0.017	0.060 ± 0.002	***
C 22:5 n-3 (DPA)	1.801 ± 0.025	0.465 ± 0.010	***
C 22:6 n-3 (DHA)	7.64 ± 0.09	5.46 ± 0.08	***
SFA	25.75 ± 0.08	18.97 ± 0.12	***
MUFA	37.34 ± 0.39	47.43 ± 0.03	***
PUFA	36.90 ± 0.31	33.59 ± 0.10	***
n-3	25.21 ± 0.22	15.71 ± 0.12	***
n-6	11.69 ± 0.09	17.88 ± 0.02	***

*** *p* < 0.001, ** *p* < 0.01, ND—not detected.

**Table 3:** Chemical composition and colour of raw wild (*n* = 12) and farmed (*n* = 12) pikeperch (mean values ± standard error).

Attribute	Wild Pikeperch	Farmed Pikeperch	*p* Value
Moisture (%)	78.88 ± 0.08	76.70 ± 0.22	***
Fat (%)	0.40 ± 0.02	1.09 ± 0.03	***
Protein (%)	19.29 ± 0.12	20.82 ± 0.21	***
Ash (%)	1.21 ± 0.01	1.09 ± 0.01	***
pH	6.69 ± 0.02	6.51 ± 0.01	***
Expressible moisture (%)	4.60 ± 0.21	4.52 ± 0.18	NS
L*	35.03 ± 0.29	35.59 ± 0.42	NS
a*	0.46 ± 0.08	−0.33 ± 0.04	***
b*	−0.99 ± 0.11	−0.98 ± 0.12	NS
C	1.25 ± 0.09	1.12 ± 0.10	NS
h	57.4 ± 4.3	68.0 ± 3.6	NS

*** *p* < 0.001, NS—non-significant differences (*p* > 0.05).

**Table 4:** Fatty acid composition of raw wild (*n* = 12) and farmed (*n* = 12) pikeperch (mean values ± standard error).

Fatty Acid	Proportion (% of Total)			Concentration (mg/100 g Wet Tissue)		
	Wild Pikeperch	Farmed Pikeperch	*p* Value	Wild Pikeperch	Farmed Pikeperch	*p* Value
Saturated fatty acids (SFA)						
C 12:0	0.185 ± 0.015	0.036 ± 0.001	***	0.504 ± 0.034	0.273 ± 0.011	***
C 14:0	4.308 ± 0.187	3.344 ± 0.035	***	11.94 ± 0.76	25.38 ± 0.65	***
C 15:0	1.066 ± 0.061	0.288 ± 0.003	***	2.94 ± 0.20	2.19 ± 0.06	**
C 16:0	22.23 ± 0.69	14.91 ± 0.51	***	62.10 ± 4.17	113.08 ± 4.54	***
C 17:0	0.919 ± 0.044	0.160 ± 0.001	***	2.60 ± 0.24	1.22 ± 0.04	***
C 18:0	4.034 ± 0.249	1.477 ± 0.012	***	11.42 ± 1.16	11.22 ± 0.31	NS
C 20:0	0.153 ± 0.005	0.162 ± 0.003	NS	0.427 ± 0.026	1.23 ± 0.04	***
Monounsaturated fatty acids (MUFA)						
C 14:1	0.189 ± 0.014	0.113 ± 0.005	***	0.517 ± 0.039	0.857 ± 0.034	***
C 16:1	9.099 ± 1.483	6.426 ± 0.160	*	25.58 ± 4.13	48.57 ± 0.97	***
C 17:1	1.312 ± 0.072	0.334 ± 0.003	***	3.57 ± 0.14	2.53 ± 0.07	***
C 18:1 cis9	16.93 ± 0.59	35.99 ± 0.23	***	46.39 ± 1.68	273.60 ± 7.79	***
C 18:1 cis11	4.310 ± 0.191	2.795 ± 0.042	***	11.79 ± 0.49	21.23 ± 0.63	***
C 20:1 n-9	0.514 ± 0.023	4.268 ±0.098	***	1.41 ± 0.06	32.41 ± 1.10	***
C 22:1 n-11	ND	1.833 ± 0.039	***	ND	13.94 ± 0.51	***
C 22:1 n-9	ND	0.318 ± 0.006	***	ND	2.42 ± 0.08	***
Polyunsaturated fatty acids (PUFA)						
C 18:2 n-6	4.608 ±0.232	11.896 ± 0.166	***	12.55 ± 0.51	90.49 ± 3.02	***
C 18:3 n-6	0.413 ± 0.017	0.169 ± 0.004	***	1.13 ± 0.05	1.29 ± 0.05	NS
C 18:3 n-3	4.233 ± 0.248	3.461 ± 0.044	**	11.59 ± 0.68	26.34 ± 0.89	***
C 18:4 n-3	0.939 ± 0.068	0.653 ± 0.007	***	2.60 ± 0.22	4.96 ± 0.16	***
C 20:2 n-6	0.327 ± 0.014	0.651 ± 0.007	***	0.91 ± 0.07	4.95 ± 0.16	***
C 20:3 n-6	0.327 ± 0.013	0.152 ± 0.003	***	0.90 ± 0.04	1.16 ± 0.04	***
C 20:4 n-6	4.200 ± 0.141	0.254 ± 0.006	***	11.77 ± 0.83	1.93 ± 0.07	***
C 20:3 n-3	0.477 ± 0.027	0.323 ± 0.003	***	1.33 ± 0.11	2.46 ± 0.08	***
C 20:4 n-3	1.142 ± 0.072	0.497 ± 0.005	***	3.21 ± 0.30	3.78 ± 0.12	NS
C 20:5 n-3 (EPA)	5.703 ± 0.187	2.561 ±0.048	***	16.01 ± 1.15	19.48 ± 0.66	*
C 22:5 n-6	0.736 ± 0.054	0.105 ± 0.004	***	2.10 ± 0.23	0.80 ± 0.04	***
C 22:5 n-3 (DPA)	1.689 ± 0.067	0.812 ± 0.007	***	4.68 ± 0.28	6.18 ± 0.18	***
C 22:6 n-3 (DHA)	9.957 ± 0.515	6.004 ± 0.089	***	28.30 ± 2.65	45.64 ± 1.41	***
SFA	32.90 ± 0.88	20.38 ± 0.50	***	91.92 ± 6.08	154.59 ± 5.07	***
MUFA	32.35 ± 1.35	52.08 ± 0.30	***	89.25 ± 4.93	395.56 ± 10.14	***
PUFA	34.75 ±0.78	27.54 ± 0.34	***	97.08 ± 6.04	209.47 ± 6.69	***
n-3	24.14 ± 0.64	14.31 ± 0.18	***	67.71 ± 4.66	108.84 ± 3.41	***
n-6	10.61 ± 0.24	13.23 ± 0.18	***	29.37 ± 1.42	100.63 ± 3.33	***
n-3/n-6	2.3	1.1				
n-6/n-3	0.4	0.9				

*** *p* < 0.001, ** *p* < 0.01, * *p* < 0.05, NS—non-significant differences (*p* > 0.05).

**Table 5:** The influence of life environment on the composition and colour of sous-vide (65 °C, 40 min) pikeperch fillets (mean values ± standard error).

Attribute	Wild Pikeperch	Farmed Pikeperch	*p* Value
Moisture (%)	74.37 ± 0.34	75.33 ± 0.40	NS
Fat (%)	0.52 ± 0.04	1.36 ± 0.05	***
Protein (%)	23.85 ± 0.33	22.10 ± 0.38	***
Ash (%)	1.20 ± 0.03	1.17 ± 0.01	NS
Cooking loss (%)	22.19 ± 0.66	19.98 ± 0.66	NS
L*	67.43 ± 1.01	73.85 ± 0.80	***
a*	−0.34 ± 0.15	0.52 ± 0.36	*
b*	11.25 ± 0.35	12.17 ± 0.44	NS
C	11.27 ± 0.35	12.24 ± 0.44	NS
h	86.94 ± 0.40	85.04 ± 0.98	NS
Sensory quality (points from 1 to 5)			
Colour	4.86 ± 0.06	4.96 ± 0.04	NS
Aroma	4.61 ± 0.08	4.58 ± 0.10	NS
Texture	4.58 ± 0.11	4.25 ± 0.12	NS
Juiciness	4.64 ± 0.11	4.42 ± 0.15	NS
Taste	4.75 ± 0.07	4.79 ± 0.08	NS

*** *p* < 0.001, * *p* < 0.05; NS—non-significant differences (*p* > 0.05).

**Table 6:** Fatty acid composition of sous-vide wild (*n* = 12) and farmed (*n* = 12) pikeperch fillets (mean values ± standard error).

Fatty Acid	Proportion (% of Total)			Concentration (mg/100 g Wet Tissue)		
	Wild Pikeperch	Farmed Pikeperch	*p* Value	Wild Pikeperch	Farmed Pikeperch	*p* Value
Saturated fatty acids (SFA)						
C 12:0	0.178 ± 0.018	0.034 ± 0.001	**	0.657 ± 0.110	0.328 ± 0.014	*
C 14:0	3.78 ± 0.07	3.40 ± 0.06	**	13.74 ± 1.22	32.31 ± 0.67	***
C 15:0	0.888 ± 0.021	0.295 ± 0.004	**	3.22 ± 0.27	2.81 ± 0.08	NS
C 16:0	19.33 ± 0.55	15.49 ± 0.26	***	70.9 ± 7.5	147.4 ± 3.1	***
C 17:0	0.878 ± 0.045	0.160 ± 0.002	**	3.23 ± 0.36	1.53 ± 0.06	**
C 18:0	3.430 ± 0.134	1.427 ± 0.043	**	12.66 ± 1.51	13.62 ± 0.82	NS
C 20:0	0.147 ± 0.001	0.155 ± 0.006	NS	0.535 ± 0.046	1.474 ± 0.079	***
Monounsaturated fatty acids (MUFA)						
C 14:1	0.167 ± 0.013	0.124 ± 0.012	*	0.59 ± 0.04	1.17 ± 0.08	***
C 16:1	11.63 ± 0.90	6.15 ± 0.32	**	41.34 ± 2.91	58.42 ± 2.62	**
C 17:1	1.23 ± 0.06	0.348 ± 0.010	***	4.40 ± 0.32	3.31 ± 0.11	*
C 18:1 cis9	16.04 ± 0.67	36.37 ± 0.15	***	58.18 ± 5.56	346.7 ± 13.1	***
C 18:1 cis11	3.92 ± 0.17	2.84 ± 0.02	***	14.13 ± 1.16	27.08 ± 0.97	***
C 20:1 n-9	0.529 ± 0.016	4.09 ± 0.15	*	1.94 ± 0.21	39.05 ± 2.30	***
C 22:1 n-11	ND	1.77 ± 0.08	***	ND	16.92 ±1.13	***
C 22:1 n-9	ND	0.304 ± 0.009	***	ND	2.90 ± 0.18	***
Polyunsaturated fatty acids (PUFA)						
C 18:2 n-6	4.55 ± 0.27	11.94 ± 0.20	***	16.29 ± 1.18	113.99 ± 5.92	***
C 18:3 n-6	0.398 ± 0.018	0.173 ± 0.002	*	1.43 ± 0.09	1.65 ± 0.05	NS
C 18:3 n-3	4.31 ± 0.26	3.47 ± 0.07	*	15.31 ± 0.74	33.17 ± 1.78	***
C 18:4 n-3	1.02 ± 0.07	0.649 ± 0.007	*	3.65 ± 0.34	6.19 ± 0.28	***
C 20:2 n-6	0.361 ± 0.016	0.651 ± 0.010	***	1.32 ± 0.12	6.21 ± 0.30	***
C 20:3 n-6	0.329 ± 0.010	0.150 ± 0.002	*	1.18 ± 0.09	1.43 ± 0.06	NS
C 20:4 n-6	4.44 ± 0.321	0.236 ± 0.005	***	16.34 ± 1.76	2.25 ± 0.12	***
C 20:3 n-3	0.529 ± 0.027	0.22 ± 0.007	***	1.92 ± 0.18	3.06 ± 0.16	**
C 20:4 n-3	1.26 ± 0.12	0.496 ± 0.009	*	4.59 ± 0.59	4.73 ± 0.24	NS
C 20:5 n-3 (EPA)	6.22 ± 0.44	2.45 ± 0.06	*	22.85 ± 2.65	23.41 ± 1.40	NS
C 22:5 n-6	0.957 ± 0.105	0.106 ± 0.004	*	3.55 ± 0.49	1.00 ± 0.04	**
C 22:5 n-3 (DPA)	1.90 ± 0.09	0.790 ± 0.019	*	6.86 ± 0.55	7.55 ± 0.45	NS
C 22:6 n-3 (DHA)	11.58 ± 0.98	5.60 ± 0.05	**	42.75 ± 5.28	53.40 ± 2.36	NS
SFA	28.63 ± 0.65	20.96 ± 0.31	***	104.9 ± 10.8	199.5 ± 4.7	***
MUFA	33.51 ± 1.68	52.00 ± 0.34	***	120.6 ± 9.9	495.5 ± 17.6	***
PUFA	37.86 ± 1.57	27.04 ± 0.42	***	138.1 ± 12.9	258.1 ± 13.1	***
n-3	26.82 ± 1.50	13.78 ± 0.22	***	97.9 ± 9.9	131.5 ± 6.6	*
n-6	11.04 ± 0.15	13.26 ± 0.21	***	1.06 ± 0.09	3.00 ± 0.11	***
n-3/n-6	2.43	1.04				
n-6/n-3	0.41	0.96				

*** *p* < 0.001, ** *p* < 0.01, * *p* < 0.05, NS—non-significant differences (*p* > 0.05).

## Data Availability

The datasets generated for this study are available on request to the corresponding author.
